# Food intake regulating-neuropeptides are expressed and regulated through pregnancy and following food restriction in rat placenta

**DOI:** 10.1186/1477-7827-6-14

**Published:** 2008-04-02

**Authors:** Jorge E Caminos, Susana B Bravo, C Ruth González, Maria F Garcés, Libia A Cepeda, Adriana C González, Fernando Cordido, Miguel López, Carlos Diéguez

**Affiliations:** 1 Department of Physiology, School of Medicine, University of Santiago de Compostela, Santiago de Compostela, Spain; 2 Department of Physiology and Genetic Institute, Faculty of Medicine, National University of Colombia. Bogotá, Colombia; 3 Endocrine Department, Hospital Juan Canalejo, A Coruña, Spain; 4 CIBER of Obesity and Nutrition, Instituto Salud Carlos III, Santiago de Compostela, Spain

## Abstract

**Background:**

Neuropeptide Y (NPY), agouti related peptide (AgRP), cocaine and amphetamine-regulated transcript (CART) and melanocortins, the products of the proopiomelanocortin (POMC), are hypothalamic peptides involved in feeding regulation and energy homeostasis. Recent evidence has demonstrated their expression in rat and human placenta.

**Methods:**

In the current study, we have investigated the expression of those neuropeptides in the rat placenta by real-time PCR using a model of maternal food restriction.

**Results:**

Our results showed that placental-derived neuropeptides were regulated through pregnancy and following food restriction.

**Conclusion:**

These data could indicate that placental-derived neuropeptides represent a local regulatory circuit that may fine-tune control of energy balance during pregnancy.

## Background

The regulation of body weight is carried out by a complex inter-organ circuit connecting the periphery and the brain, where neurons in the hypothalamus and brainstem exert potent effects on feeding and energy expenditure [1,2]. Short-acting and long-term body-weight regulating signals mainly originate from: 1) the adipose tissue, such as leptin, and interleukin 6 (IL-6); 2) the pancreas, like insulin or amylin; 3) the gastrointestinal tract such as ghrelin, glucagon-like peptide-1 (GLP-1), peptide YY (PYY), cholecystokinin (CCK) and neuropeptide W (NPW); 4) the sensory vagus nerve [2]. These neural and humoral signals interact at the brain level with widely-located target receptors related with the nutritional state, metabolism and reproduction [2,3].

Hypothalamic neuropeptide expression and regulation are stimulated by inputs from peripherally-derived hormonal and nutrient-related signals essential for the central control of energy homeostasis in mammals. Neuropeptide Y (NPY), melanocortins (POMC-derived products), agouti related peptide (AgRP), cocaine and amphetamine regulated transcript (CART), melanin concentrating hormone (MCH) and orexins, have been characterized in both rodent models and humans, by having either direct or indirect effects in the feedback loop of body weight regulation and reproduction [4,5].

NPY and AgRP are two anabolic neuropeptides that promote weight gain by reducing energy expenditure and stimulating food intake. They are coexpressed in arcuate nucleus (ARC) neurons, which are inhibited by insulin and leptin [4,6]. In contrast, CART and POMC, also coexpressed in the ARC neurons, are stimulated by inputs from insulin and leptin and these catabolic neuronal pathways act to reduce feeding and increase energy expenditure [4,6].

Besides their role in food intake control, hypothalamic neuropeptides have also been identified in female reproductive tissues, such as the placenta and appear to have an important role in the physiology of pregnancy [7-14]. It is interesting to note that the placenta may play an important role not only in reproduction but is also an important site for translating inputs from diverse hormonal signals that powerfully influence energy balance within the fetus [15,16]. The aim of this study was to evaluate the ontogenic mRNA expression pattern of NPY, AgRP, POMC, and CART throughout pregnancy in the rat placenta. In addition, we have determined the effects of chronic food restriction on their expression levels.

## Methods

### Animals

Pregnant female rats of the Sprague Dawley strain aged between 10–12 weeks (bred in the Animalario General USC; Santiago de Compostela, Spain), were housed in a temperature-regulated room with a 12 h light/12 h dark cycle, with tap water and standard rat chow *ad libitum *or a food-restricted diet (see below). At the end of the study period, animals were sacrificed, hypothalami and placentas were removed from each mother, and snap-frozen in liquid nitrogen for RNA extraction and real time semi-quantitative RT-PCR. All experiments and procedures in this study were carried out according to a protocol approved by the Ethics Committee of the University of Santiago de Compostela in accordance with the European Union normative for the care and use of experimental animals.

### Maternal chronic food-restriction

A maternal chronic food-restriction model was used throughout pregnancy to study the effect of long-term undernutrition on placental neuropeptide mRNA expression as previously described [17]. Briefly, virgin rats were mated on the day of proestrus and the day on which spermatozoa were present in vaginal smear, was designated gestational day 1. The pregnant rats were divided into two dietary groups: pregnant rats fed *ad libitum *and food-restricted group of pregnant rats fed 30% of *ad libitum *intake. Food-restricted animals were fed every day at 18:00 hours. Rats were sacrificed at gestational days 12, 16 and 21. We used 9 hypothalami and placentas per experimental group, extracted from 9 different mothers. All the samples were analyzed individually and samples were not pooled.

### RNA Isolation and real-time RT-PCR

Placental neuropeptide gene expression was analyzed by reverse transcription polymerase chain reaction (RT-PCR) as previously validated [18-20]. Total RNA was isolated using Trizol according to the manufacturer's protocol (Invitrogen, CA). First-strand cDNA was synthesized from 2 μg of total RNA by random primer reverse transcription. A negative control without MMLV reverse transcriptase was used to ensure specificity of the PCR amplification. The resulting cDNA was subjected to PCR amplification using sense and antisense primers specific for rat AgRP, CART, NPY and POMC mRNAs (Table 1). Primer pairs were designed overlapping on different exons to prevent amplification from any contaminated genomic DNA (data not shown). To verify the identity of amplified cDNAs, PCR products were electrophoresed on a 1.5% agarose gel, which yielded DNA fragments of the expected length for all mRNAs and were confirmed by sequencing (data not shown). Rat hypoxanthine guanine phosphoribosyl transferase (HPRT) was used as a control housekeeping gene. PCR was performed by real-time in LightCycler real-time PCR machine 2 (Roche Diagnostics, Germany) and the software version 3.5, according to the method described elsewhere [19-21]. Briefly, the PCR protocol consisted of initial denaturation at 96°C for 5 min, followed by 35 cycles of denaturation (96°C for 2 s), annealing (62°C for 15 s) and elongation (72°C for 15 s). This was followed by melting curve analysis consisting of 1 cycle at 95°C for 30 s, 60°C for 15 s and a temperature rise to 85°C at a slope of 0.2°C/s with continuous measurement of fluorescence. The primers specify was also assured by the melting curve of each gene. All samples were standardized against HPRT by using the 2ΔΔct method [19,22].

**Table 1:** Primers used for real-time RT-PCR

**mRNA**	**Primer Sequence (5'-3')**		**Product size (bp)**	**Accession Number**
NPY	Forward	cgctctgcgacactacatca	157	NM_012614
	Reverse	tttcatttcccatcaccaca		
AgRP	Forward	tgtgggccctttattagacc	156	XM_574228
	Reverse	ccatatggacccccaatgt		
POMC	Forward	tccatagacgtgtggagctg	174	NM_139326
	Reverse	gacgtacttccggggatttt		
CART	Forward	gccctggacatctactctgc	201	NM_017110
	Reverse	cactgcgcactgctctcc		
HPRT	Forward	cagtcccagcgtcgtgatta	137	NM_012583
	Reverse	agcaagtctttcagtcctgtc		

### Statistical analysis

Statistical analyzes were performed using GraphPad Instat (version 3.05). All group values were expressed as mean ± SEM. Differences between groups were analyzed by using one-way analysis of variance (ANOVA) or Student's t-test. P < 0.05 was considered significant.

## Results

### NPY mRNA expression is down-regulated by food restriction in the rat placenta

**Figure 1:**
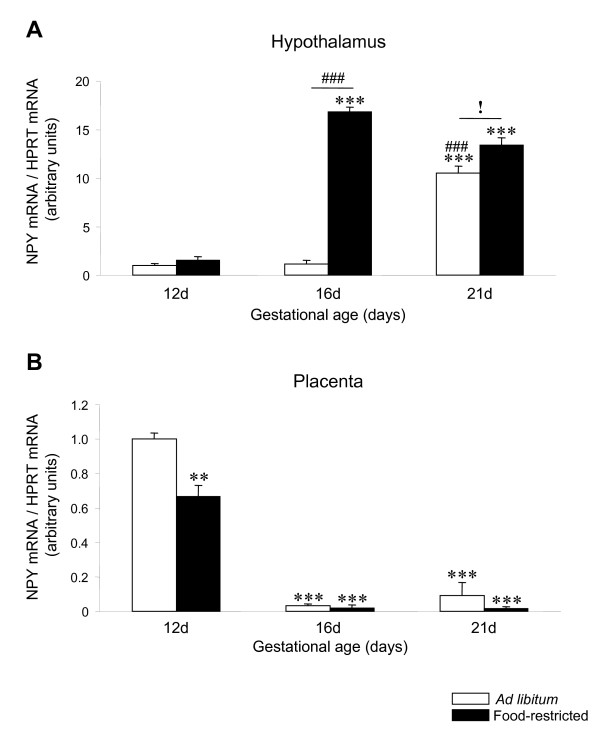
**NPY expression in hypothalamus and placenta of *ad libitum *fed and food-restricted rats**. Relative NPY mRNA levels in (A) hypothalamus and (B) placenta from *ad libitum *fed or food-restricted female rats at different pregnancy stages (12, 16 and 21 days). Relative mRNA levels were normalized to *ad libitum *fed (control) as 1. **: P < 0.01 *vs. ad libitum *12d; ***: P < 0.001 *vs. ad libitum *12d; ###: P < 0.001 *vs. ad libitum *16d; !: P < 0.05 *ad libitum *21d *vs*. food-restricted 21d.

Firstly, we studied the hypothalamic mRNA levels of NPY during pregnancy and after food restriction. Our data showed that the transcriptional expression of NPY is increased at the latest pregnancy stage (21 days; P < 0.001) with food restriction inducing a further increase at both 16 and 21 days of pregnancy (P < 0.001 and P < 0.05, respectively) (Figure 1A). Next, we evaluated the transcriptional expression of NPY in rat placenta; our results showed that placental mRNA levels of NPY were significantly higher during the initial period of gestation studied (12 days), and decreased significantly (P < 0.001) with gestational age (Figure 1B). We also studied the effect of food restriction on placental mRNA expression of NPY and these data showed that the expression profile of NPY was decreased by food restriction only at day 12 (P < 0.01), and remain unchanged between 16 to 21 days, compared to the *ad libitum *group (Figure 1B).

### AgRP mRNA expression is up-regulated by food restriction in the rat placenta

**Figure 2:**
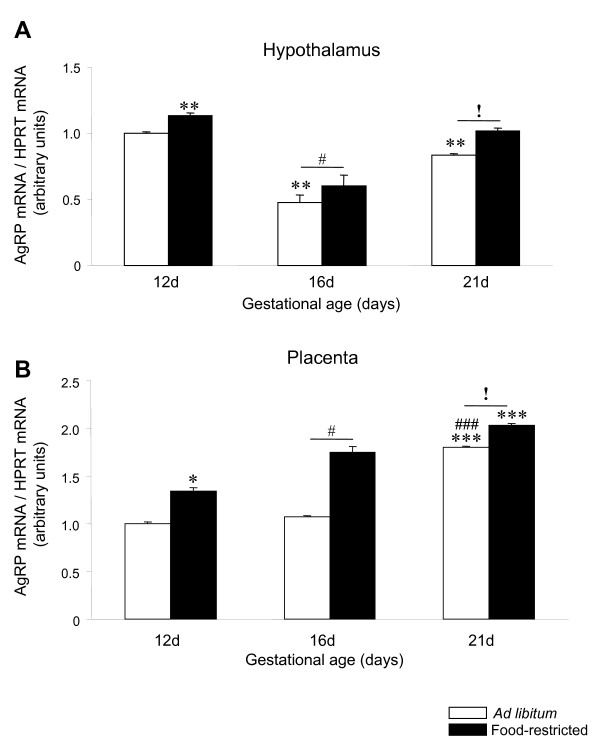
**AgRP expression in hypothalamus and placenta of *ad libitum *fed and food-restricted rats**. Relative AgRP mRNA levels in (A) hypothalamus and (B) placenta from *ad libitum *fed or food-restricted female rats at different pregnancy stages (12, 16 and 21 days). Relative mRNA levels were normalized to *ad libitum *fed (control) as 1. *: P < 0.05 *vs.ad libitum *12d; **: P < 0.01 *vs. ad libitum *12d; ***: P < 0.001 *vs. ad libitum *12d; #: P < 0.05 *vs. ad libitum *16d; ###: P < 0.001 *vs. ad libitum *16d; !: P < 0.05 *ad libitum *21d *vs*. food-restricted 21d.

Throughout pregnancy, hypothalamic mRNA expression levels of AgRP were decreased (P < 0.01) at 16 and 21 days of gestation compared to 12 days and these levels were increased by food restriction (P < 0.05 and P < 0.01) at all days of gestation studied compared to the *ad libitum *(Figure 2A). In rat placenta, throughout the same experimental period, the expression mRNA profile of AgRP was opposite to NPY (Figure 2B). Placental AgRP expression remained unchanged between 12 and 16 days of gestation reaching the highest levels at the end of the gestation period (P < 0.001)(Figure 2B). Food restriction induced a significant and progressive increase (P < 0.05) in placenta AgRP mRNA expression at all studied time points.

### POMC mRNA expression shows a biphasic response to food restriction during pregnancy in rat placenta

**Figure 3:**
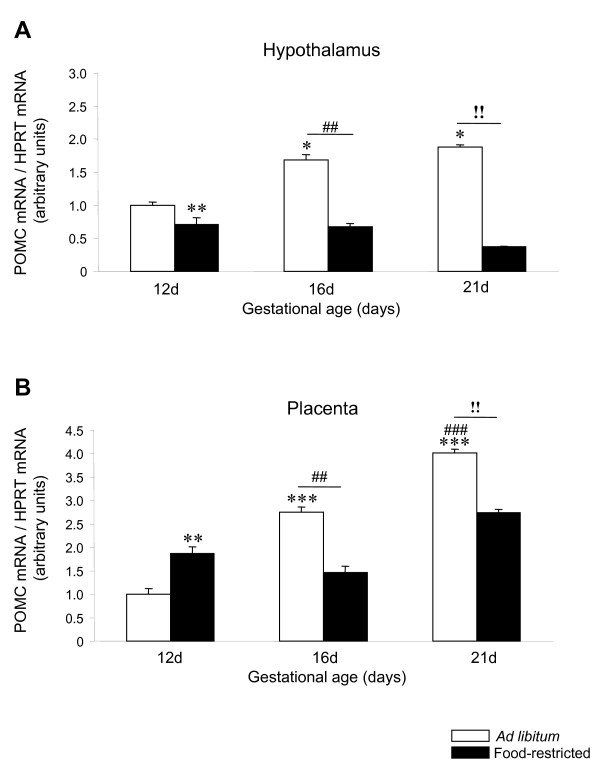
**POMC expression in hypothalamus and placenta of *ad libitum *fed and food-restricted rats**. Relative POMC mRNA levels in (A) hypothalamus and (B) placenta from *ad libitum *fed or food-restricted female rats at different pregnancy stages (12, 16 and 21 days). Relative mRNA levels were normalized to *ad libitum *fed (control) as 1. *: P < 0.05 *vs. ad libitum *12d; **: P < 0.01 *vs. ad libitum *12d; ***: P < 0.001 *vs. ad libitum *12d; ##: P < 0.01 *vs. ad libitum *16d; ###: P < 0.001 *vs. ad libitum *16d; !!: P < 0.01 *ad libitum *21d *vs*. food-restricted 21d.

Hypothalamic POMC mRNA expression was increased during pregnancy (P < 0.05) and decreased by food restriction (P < 0.01) at 12, 16 and 21 days of gestation (Figure 3A). Placental POMC mRNA expression showed a significantly (P < 0.001) increasing trend of expression from 12 to 21 days of gestation in rats fed *ad libitum *(Figure 3B). Placental POMC expression was significantly (P < 0.01) lower in fed *ad libitum *rats than in food-restricted group at 12 days. Conversely, significantly (P < 0.01) lower levels of POMC expression were detected in the food-restricted group at compared to fed *ad libitum *experimental group from 16 to 21 days of gestation.

### CART mRNA expression is down-regulated by food restriction in rat placenta

**Figure 4:**
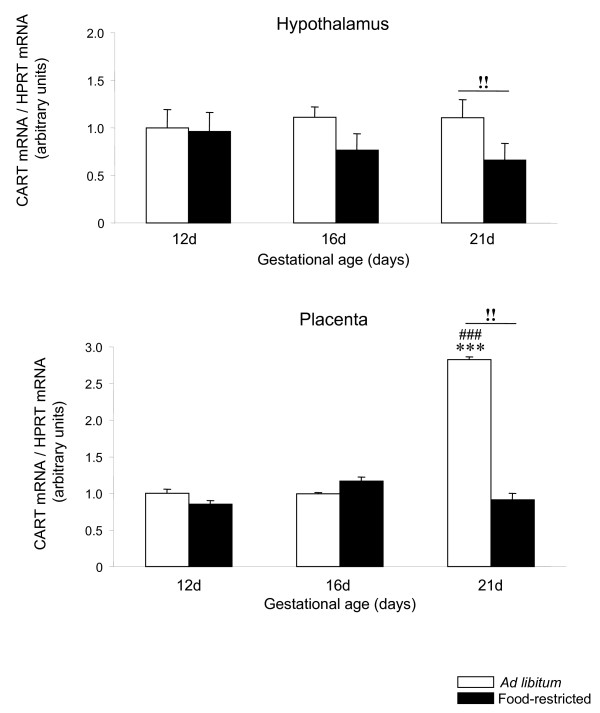
**CART expression in hypothalamus and placenta of *ad libitum *fed and food-restricted rats**. Relative CART mRNA levels in (A) hypothalamus and (B) placenta from *ad libitum *fed or food-restricted female rats at different pregnancy stages (12, 16 and 21 days). Relative mRNA levels were normalized to *ad libitum *fed (control) as 1. ***: P < 0.001 *vs. ad libitum *12d; ###: P < 0.001 *vs. ad libitum *16d; !!: P < 0.01 *ad libitum *21d *vs*. food-restricted 21d.

Hypothalamic CART mRNA expression did not show any change during the different stages of pregnancy and was decreased by food restriction (P < 0.01) at 21 days of gestation (Figure 4A). Placental CART mRNA expression did not change significantly from 12 to 16 days, showing the highest levels (P < 0.001) at 21 days of gestational age. In addition, CART mRNA expression remained unchanged throughout pregnancy from 12 to 16 days of pregnancy in food-restricted rats. Conversely, at 21 days of gestational age mRNA levels of CART were significantly lower in food-restricted rats than in fed *ad libitum *rats (P < 0.01) (Figure 4B).

## Discussion

Although the expression of feeding neuropeptides in placenta is well established, little is known about the regulation of their expression during pregnancy and by nutritional status. In this study, we investigated the placental mRNA expression levels of NPY, AgRP, CART and POMC throughout pregnancy (from day 12 to day 21) in fed and food-restricted rats.

Fetal development is critically determined by the availability and flux of nutrients and oxygen across the placenta during pregnancy. The placenta tissue is an active endocrine organ involved in the control of not only metabolism and energy balance, but also of other relevant body functions including reproduction [23,24]. Previous studies have demonstrated the placental expression of hormones, such as ghrelin and leptin [25-27], as well as neuropeptides such as NPY, AgRP, CART and POMC [7-11] are involved in the regulation of energy homeostasis. However, despite these data, both the physiogical relevance and the regulation of these molecules in placental tissues remain unclear.

In the hypothalamus, food restriction induces opposite and compensatory changes in neuropeptide expression. Thus, orexigenic neuropeptides such as AgRP and NPY are up-regulated by decreased food availability whilst anorexigenic neuropeptides such as CART and POMC are downregulated under the same conditions. Interestingly, AgRP, CART and POMC follow the same expression pattern in placental tissues but NPY is down-regulated in food-restricted rats. The reason for this discrepancy is unclear, but it could be related to an altered hormonal milieu after food restriction. In this sense, recent data from our group demonstrates that pregnancy hormones, such as prolactin, play a major role on hypothalamic NPY expression [28]. Whether this interaction is present in placental tissues will merit further investigation. In addition, these data support the fact that contrary to the pattern in the ARC, AgRP and NPY are probably coexpressed by different cell populations in placental tissue and may be modulated by different signals.

Whatever the case, the discrepancies in NPY expression pattern suggest that an anabolic role for placental NPY in states of increased energy demand is not clear and this possibility merits further investigation. The data from AgRP, CART and POMC suggest that their expression may be under the same, or at least, a similar transcriptional control to that in the hypothalamus. Whether placental neuropeptide protein levels correlate with mRNA expression, as in the hypothalamus, will also merit further investigation.

## Conclusion

In summary, we demonstrate that central signals involved in energy homeostasis in the hypothalamus are also expressed and modulated by nutritional status in the rat placenta. These neuropeptides display a specific ontogenic expression pattern which change over the entire gestational range. They are also affected by chronic food restriction. Altogether, these data suggest that neuropeptides could play a role in the homeostatic response to energy availability in rat placenta.

## Competing interests

With respect to the disclosure of any financial interest, we state that none of the authors of this manuscript have any financial interest that has influenced the results or interpretation of this manuscript.

## Authors' contributions

JEC, SBB, CRG, MFG, LAC, ACG performed the experiments. JEC, SBB, CRG, FC, ML and CD edited and reviewed the manuscript and figures. JEC, SBB, ML and CD wrote the manuscript.
